# Non-Skeletal Roles of Vitamin D in Skin, Gut, and Cardiovascular Disease: Focus on Epithelial Barrier Function and Immune Regulation in Chronic Disease

**DOI:** 10.3390/ijms26178520

**Published:** 2025-09-02

**Authors:** Teresa Grieco, Giovanni Paolino, Elisa Moliterni, Camilla Chello, Alvise Sernicola, Maria Luisa Brandi, Colin Gerard Egan, Mariangela Morelli, Fabrizio Nannipieri, Santina Battaglia, Marina Accoto, Erika Tirotta, Silvia Trasciatti, Silvano Bonaretti, Camilla Calvieri, Giovanni Pellacani, Stefano Calvieri

**Affiliations:** 1 Dermatology Clinic, Department of Clinical Internal, Anesthesiological and Cardiovascular Sciences, Sapienza University of Rome, 00185 Rome, Italy; teresa.grieco@uniroma1.it (T.G.); elisa.moliterni@gmail.com (E.M.); c.chello@unicampus.it (C.C.); alvise.sernicola@uniroma1.it (A.S.); giovanni.pellacani@uniroma1.it (G.P.); stefano.calvieri@uniroma1.it (S.C.); 2 Unit of Dermatology and Cosmetology, IRCCS Ospedale San Raffaele, 20132 Milan, Italy; 3 Fondazione FIRMO Onlus (Fondazione Italiana Ricerca sulle Malattie dell’Osso), 50129 Florence, Italy; marialuisa@marialuisabrandi.it; 4 CE Medical Writing SRLS, 56021 Pisa, Italy; colingegan@gmail.com; 5 Fondazione Pisana per la Scienza, 56017 Pisa, Italy; mmorelli190977@gmail.com; 6 Clinical Research, Abiogen Pharma, 56121 Pisa, Italy; fabrizio.nannipieri@abiogen.it (F.N.); santina.battaglia@abiogen.it (S.B.); marina.accoto@abiogen.it (M.A.); erika.tirotta@abiogen.it (E.T.); 7 Galileo Research Srl, 56019 Pisa, Italy; silvia.trasciatti@galileoresearch.it (S.T.); silvano.bonaretti@galileoresearch.it (S.B.); 8 Department of Clinical Internal, Anesthesiologic and Cardiovascular Sciences, Sapienza University of Rome, Piazzale Aldo Moro 5, 00185 Rome, Italy; camilla.calvieri@yahoo.it

**Keywords:** vitamin D, epithelial barrier, inflammation, tight junctions, atopic dermatitis, inflammatory bowel disease, cardiovascular disease, immune homeostasis

## Abstract

Vitamin D is increasingly recognized as a key regulator of epithelial barrier integrity and mucosal immune homeostasis, with implications extending far beyond skeletal health. Through the vitamin D receptor (VDR), vitamin D regulates epithelial cohesion, innate immune responses, and tight-junction gene expression. This review explores the multifactorial role of vitamin D in modulating inflammation and preserving tissue barriers, with particular emphasis on its effects on tight junction (TJ) regulation and disease states characterized by barrier dysfunction, namely atopic dermatitis, psoriasis, inflammatory bowel disease (IBD), and celiac disease. In these settings, vitamin D/VDR signaling exerts protective actions by enhancing barrier structure, suppressing Th1/Th17-driven inflammation, modulating the gut and skin microbiome, and promoting epithelial repair. Animal studies and clinical data suggest that vitamin D supplementation can restore TJ expression, reduce disease activity, and improve clinical outcomes in both intestinal and dermatologic diseases. In the cardiovascular system, the role of vitamin D remains complex. While vitamin D influences endothelial function, insulin sensitivity, and systemic inflammation, supplementation trials yield mixed results, indicating a need for individualized approaches. Overall, this review synthesizes mechanistic, translational, and clinical data supporting vitamin D as a crucial modulator of barrier integrity and inflammation. These findings highlight its therapeutic relevance in chronic diseases characterized by immune dysregulation and epithelial disruption.

## 1. Introduction

Vitamin D is a secosteroid hormone primarily synthesized in the skin upon exposure to ultraviolet B (UVB) radiation or obtained through dietary sources, including fortified foods and supplements. It undergoes hydroxylation in the liver to form 25-hydroxyvitamin D [25(OH)D], the main circulating form, followed by further conversion in the kidneys to its active form, 1,25-dihydroxyvitamin D [1,25(OH)2D] [1]. While its classical role in calcium and phosphate homeostasis and bone metabolism is well-documented, an expanding body of research has revealed its far-reaching effects on various physiological processes, including immune regulation, cardiovascular function, and epithelial barrier integrity [2]. The presence of vitamin D receptors (VDRs) in nearly all nucleated cells suggests that vitamin D functions as a crucial modulator of cellular activity across multiple organ systems [3]. VDRs are highly expressed in immune cells, endothelial tissues, and epithelial barriers, indicating a broad functional role in maintaining tissue homeostasis and regulating inflammatory responses. Vitamin D is also recognized to influence the transcription of more than 200 genes involved in cell differentiation, immune defense, and metabolism, showing its broad systemic effects [4]. Recent epidemiological studies have linked vitamin D deficiency with an increased risk of chronic diseases beyond skeletal disorders, including autoimmune diseases, cardiovascular disease (CVD), metabolic syndrome, neurodegenerative disorders, and inflammatory bowel disease (IBD) [5]. This has led to a growing interest in the potential of vitamin D supplementation as a preventive or adjunctive therapeutic strategy in non-skeletal conditions. However, the mechanisms underlying these associations remain incompletely understood, and clinical trials evaluating vitamin D supplementation have yielded mixed results, underscoring the complexity of its systemic effects [6].

**Figure 1:**
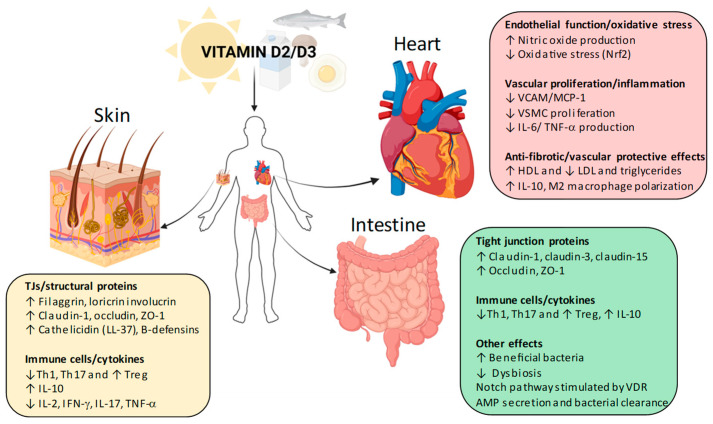
Vitamin D non-skeletal effects in skin, gut, and cardiovascular system. Schematic illustration of the pleiotropic actions of vitamin D beyond bone and mineral metabolism. In the skin, vitamin D–VDR signaling strengthens the epidermal barrier by upregulating structural proteins (filaggrin, loricrin, involucrin) and tight junction proteins (claudin-1, occludin, ZO-1), while also boosting antimicrobial peptide production (cathelicidin, β-defensins). Immune modulation includes suppression of Th1 and Th17 responses, reduced pro-inflammatory cytokines (IL-2, IFN-γ, IL-17, TNF-α), and increased Treg activity and IL-10 release. In the intestine, vitamin D enhances barrier function through increased tight junction proteins (claudin-1, claudin-3, claudin-15, occludin, ZO-1), promotes a favorable microbiota balance, stimulates the Notch pathway, and augments antimicrobial peptide secretion, thereby reducing dysbiosis and supporting mucosal immunity. In the cardiovascular system, vitamin D improves endothelial function by stimulating nitric oxide production and reducing oxidative stress via Nrf2. It limits vascular inflammation and proliferation by downregulating adhesion molecules (VCAM-1, MCP-1), vascular smooth muscle cell proliferation, and pro-inflammatory cytokines such as IL-6 and TNF-α. Additionally, vitamin D exerts anti-fibrotic and vascular-protective effects by improving lipid profiles (↑ HDL, ↓ LDL and triglycerides) and promoting anti-inflammatory macrophage polarization (IL-10, M2 phenotype). AMPs, antimicrobial peptides; HDL, high-density lipoprotein; IFN-γ, interferon-gamma; IL-2, interleukin-2; IL-6, interleukin-6; IL-10, interleukin-10; IL-17, interleukin-17; LDL, low-density lipoprotein; MCP-1, monocyte chemoattractant protein-1; NF-κB, nuclear factor kappa-light-chain-enhancer of activated B cells; NO, nitric oxide; Nrf2, nuclear factor erythroid 2–related factor 2; PMCA, plasma membrane Ca^2+^ ATPase; RAAS, renin–angiotensin–aldosterone system; TJs, tight junctions; TNF-α, tumor necrosis factor-alpha; TRPV5/6, transient receptor potential vanilloid 5/6; Treg, regulatory T cell; VCAM-1, vascular cell adhesion molecule-1; VDR, vitamin D receptor; VSMC, vascular smooth muscle cell; ZO-1, zonula occludens-1.

A significant aspect of the non-skeletal functions of vitamin D is its role in epithelial barrier integrity. In the skin, vitamin D regulates tight junction (TJ) proteins such as occludin, claudins, and zonula occludens-1 (ZO-1), which are essential for maintaining barrier function and preventing pathogen infiltration [7] (Figure 1). Similar regulatory effects have been observed in the gut, where vitamin D deficiency has been associated with increased intestinal permeability and heightened susceptibility to inflammatory diseases such as Crohn’s disease (CD) and ulcerative colitis (UC) [8] (see Figure 1). These findings suggest that vitamin D plays a protective role in maintaining epithelial defenses across different organ systems.

Furthermore, vitamin D has emerged as a key modulator of immune function. It exerts immunoregulatory effects by modulating Toll-like receptor (TLR) signaling, enhancing antimicrobial peptide production (such as cathelicidin), and promoting anti-inflammatory cytokines while inhibiting pro-inflammatory pathways [9]. This has significant implications for autoimmune diseases and chronic inflammatory conditions, where vitamin D deficiency is commonly observed and may contribute to disease pathogenesis.

In the cardiovascular system, vitamin D has been implicated in endothelial function, arterial stiffness, and the regulation of blood pressure through its effects on the renin–angiotensin–aldosterone system (RAAS) [10]. Observational studies have shown an inverse relationship between serum vitamin D levels and the incidence of hypertension, myocardial infarction, and stroke, suggesting a cardioprotective role for vitamin D [11,12] (Figure 1). However, interventional trials evaluating vitamin D supplementation for cardiovascular disease prevention have yielded inconsistent findings, highlighting the need for further research to clarify optimal dosing and target populations [13]. Finally, regarding non-skeletal effects of vitamin D, it is known that it plays a pivotal role also in malignancies. Specifically, vitamin D has been increasingly recognized for its immunomodulatory and antiproliferative properties, with potential relevance in oncology. Vitamin D receptors are expressed in melanocytes and melanoma cells, indicating a direct regulatory role [14,15,16]. Nonetheless, further prospective studies are needed to clarify its therapeutic potential and optimal supplementation strategies.

This review aims to provide a comprehensive overview of the emerging non-skeletal roles of vitamin D, with particular focus on its effects on skin integrity, cardiovascular health, gut barrier function and immune regulation. By synthesizing current evidence from clinical studies, animal models, and mechanistic research, we seek to elucidate the biological pathways through which vitamin D exerts its diverse physiological effects and explore its potential as a therapeutic target in non-skeletal diseases.

## 2. Vitamin D and the Skin Barrier: Atopic Dermatitis and Psoriasis

The skin is not only a site of vitamin D synthesis but also a target of vitamin D’s actions. Active vitamin D (calcitriol, 1,25(OH)2D) is generated locally in the skin via keratinocyte expression of CYP27B1 (1α-hydroxylase) from circulating 25(OH)D and is inactivated by CYP24A1 (24-hydroxylase) [17]. By binding to VDR in keratinocytes, calcitriol promotes differentiation, supports stratum corneum formation, and regulates the expression of key barrier proteins [18]. TJ components in the stratum granulosum, including claudins, occludin, and zonula occludens-1 (ZO-1), form the sealing barrier between keratinocytes [19]. Calcitriol has been shown to enhance the expression and membrane localization of TJ proteins such as claudin-4, claudin-7, occludin, and ZO-1 in human keratinocyte monolayers [20]. In wounded skin, increased CYP27B1 activity enhances vitamin D activation and induces antimicrobial peptides like cathelicidin (LL-37), which contribute to both antimicrobial defense and barrier repair [21]. Conversely, vitamin D deficiency or impaired VDR function can disrupt keratinocyte maturation and compromise barrier integrity, potentially contributing to inflammatory skin conditions [17]. Through these mechanisms, promoting differentiation, tightening cell junctions, and boosting antimicrobial defenses, adequate vitamin D activity is crucial for optimal skin barrier performance (Figure 1; Table 1).

### 2.1. The Regulatory Role of Vitamin D in Atopic Dermatitis

Atopic dermatitis (AD), also termed atopic eczema, represents a prevalent, chronic, and recurrent inflammatory dermatosis [22]. It is a complex disease with a spectrum of clinical presentations and combinations of symptoms [22]. A central pathogenic feature is the defective skin barrier, which leads to increased transepidermal water loss and heightened permeability to allergens, microbes, and irritants [23]. Contributing factors include altered expression of structural proteins (i.e., filaggrin, loricrin, involucrin, keratins), imbalance in protease activity, changes in pH, and ceramide loss. Together, these weaken the lipid barrier and reduce hydration [24]. Among these, filaggrin loss-of-function mutations represent the most studied genetic risk factor for AD, although their frequency varies significantly across populations, from a few percent to nearly half [25,26]. In addition, immune dysregulation plays a pivotal role, involving a disturbed balance among T helper 1 (Th1) cells, Th2, and regulatory T cells (Tregs), leading to skewed Th1/Th2 immune responses and heightened IgE-mediated allergic reactions. In AD lesions, Th2-associated cytokines, particularly interleukin (IL)-4 and IL-13, are markedly upregulated [27]. These mediators impair epidermal maturation, resulting in decreased production of filaggrin and antimicrobial peptides. Moreover, IL-31 is implicated in inducing intense itching and further suppresses keratinocyte differentiation. As the disease progresses, the immune response becomes increasingly complex, with involvement of additional cell subsets such as Th17 and Th22 cells, contributing to chronic inflammation and barrier impairment [28].

Vitamin D mediates its biological activity predominantly through the VDR, which is expressed in keratinocytes as well as in various immune cell types, including T lymphocytes and monocytes [29]. Upon binding of its active form, calcitriol, the VDR regulates the transcription of numerous genes involved in maintaining cutaneous immune balance and skin barrier integrity [30]. Among its key functions, vitamin D promotes keratinocyte maturation and enhances the expression of antimicrobial peptides (AMPs) such as cathelicidin and β-defensins, molecules that play a critical role in the innate immune defense of the skin and are often deficient in AD [30].

Vitamin D also participates in the fine-tuning of both the innate and adaptive immune response. VDR expression has been confirmed in multiple immune cell subsets, and its activation by 1,25(OH)2D has been shown to inhibit the proliferation of Th1 cells, which are typically responsible for producing cytokines like interferon-γ (IFN-γ) and IL-2, as well as for macrophage activation [31]. In parallel, vitamin D suppresses Th17 cell activity, another pro-inflammatory subset that secretes IL-17 and IL-22, both implicated in skin inflammation [29,32,33]. On the other hand, it favors the expansion and function of regulatory T cells (CD4+/CD25+), enhancing IL-10 secretion and thereby promoting an anti-inflammatory microenvironment [29]. In vitro experiments have further clarified the mechanistic role of vitamin D in skin immunity. Treatment of keratinocytes with 1,25(OH)2D has been shown to stimulate the expression of cathelicidin, an antimicrobial peptide effective against Staphylococcus aureus. This induction enhances cutaneous antimicrobial defenses and selectively downregulates cutaneous lymphocyte-associated antigen (CLA), a molecule involved in directing T lymphocytes to the skin [34,35].

Interestingly, this effect appears to be tissue-specific, as cathelicidin does not alter lymphocyte trafficking to other organs [34]. These findings provide mechanistic support for the clinical benefits of vitamin D supplementation in AD, aligning with its known roles in skin barrier regulation and immune modulation [34,35,36].

### 2.2. Clinical and Epidemiological Evidence Linking Vitamin D to AD

A growing body of clinical, epidemiological, and immunological research points to a significant association between vitamin D status and the development and severity of AD. Numerous observational studies and meta-analyses have consistently demonstrated that individuals with AD, both children and adults, exhibit lower circulating levels of 25(OH)D compared to healthy controls [37,38,39,40,41]. In a recent 2024 cross-sectional study including 681 participants aged 0–30 years, 84% of AD patients were found to have vitamin D deficiency or insufficiency, with significantly reduced levels among those with moderate-to-severe disease (EASI > 10) [37]. Notably, individuals with serum 25(OH)D levels below 25 nmol/L had a threefold increased risk of severe AD, and vitamin D concentrations inversely correlated with disease severity (r = −0.22; *p* < 0.001) [37]. This inverse relationship has been further confirmed by systematic reviews and meta-analyses.

Two reviews and a meta-analysis of 11 randomized controlled trials (RCTs, *n* = 686) demonstrated that vitamin D supplementation significantly reduced AD severity, as measured by SCORAD and EASI scores. Greater benefits were observed with daily doses of 1500–1600 IU administered for at least 12 weeks [40,41,42]. Additional studies support this correlation, showing symptom improvement and better disease control in patients with adequate vitamin D levels [36,43,44]. In particular, pediatric research suggests that sufficient vitamin D may be protective, helping to mitigate AD severity [44].

Maternal vitamin D intake during pregnancy has also been implicated in reducing the risk of atopic conditions in offspring, including wheezing disorders and eczema, indicating a possible immunoprotective role in early development [45]. Moreover, vitamin D deficiency at birth has been associated with a higher incidence of AD in infancy [46].

Despite these findings, some studies have yielded conflicting results, with reports of no significant association or even a paradoxical positive correlation between serum vitamin D levels and AD risk [47,48]. The variability in findings could be attributed to methodological heterogeneity across studies, such as differences in design, sample size, follow-up duration, and participant selection criteria, as well as to the lack of standardized supplementation protocols regarding dosage, duration, and timing. Moreover, since atopic dermatitis is influenced by numerous genetic and environmental factors, vitamin D likely represents only one of many contributing elements.

Genetic analyses have explored associations between vitamin D pathway polymorphisms and AD risk. Polymorphisms in the VDR gene, particularly BsmI, ApaI, TaqI, and A1012G, have been linked to increased disease susceptibility or severity [49,50,51]. The CYP24A1 rs2248359 C allele and related haplotypes have also been associated with more severe phenotypes [52]. In an Italian cohort, the heterozygous A1012G variant conferred protection against juvenile-onset AD (OR: 0.046, CI: 0.004–0.51, *p* = 0.012), while individuals with two or more homozygous variants in vitamin D-related genes had a significantly higher risk of early-onset severe disease and allergic sensitization (*p* = 0.0003) [51]. These variants were also linked to altered expression of TJ proteins: ZO-1 was associated with obesity (OR: 12.1, *p* = 0.045), and claudin-1 with allergic sensitization (OR: 8.23, *p* = 0.046) [51]. Notably, serum 25(OH)D levels inversely correlated with ZO-1 expression (ρ = −0.43, *p* = 0.0058) [51]. A follow-up proteomic analysis by the same authors revealed a 77.7% increase in protein content in lesional vs. perilesional biopsies (*p* < 0.001), including elevated expression of VDR, filaggrin, occludin, claudin-1, cingulin, and cathelicidin [7].

Patients with vitamin D levels below 30 ng/mL had increased expression of CYP24A (*p* = 0.054), alpha-catenin (*p* = 0.043), and haptoglobin (*p* = 0.033) [7]. In severe cases (EASI ≥ 16), higher expression levels of CYP24A, CYP27B1, filaggrin, claudin-1, and occludin were noted (all *p* < 0.05). Multivariate models confirmed strong associations between low vitamin D, altered TJ proteins, and allergic sensitization [7]. These results highlight the multifaceted role of vitamin D in maintaining skin integrity and regulating immune responses in AD (Table 2).

These findings highlight vitamin D as a potentially modifiable factor in atopic dermatitis, emphasizing its translational relevance as both a preventive target and an adjunctive therapeutic strategy.

### 2.3. The Regulatory Role of Vitamin D in Psoriasis

Psoriasis is a chronic inflammatory skin disease [69], characterized by dysregulated immune responses that result in persistent inflammation, accelerated keratinocyte proliferation, and the development of erythematous, scaly plaques [69]. Although its precise etiology remains not fully understood, psoriasis is widely regarded as a multifactorial condition driven by the interaction between genetic predisposition and environmental factors such as infections, skin trauma (Koebner phenomenon), psychological stress, smoking, and ultraviolet radiation [70]. In genetically susceptible individuals, these external stimuli may trigger aberrant immune activation, leading to chronic cutaneous inflammation and excessive epidermal turnover [71].

At the immunological level, psoriasis mainly involves T cell activation and an imbalance that favors Th1 and Th17 responses [72]. Under normal conditions, Th1 cells release pro-inflammatory cytokines such as IL-2, IFN-γ, and IL-12, while Th2 cells produce anti-inflammatory cytokines like IL-4 and IL-10. In psoriatic patients, this balance shifts toward Th1 predominance, along with an expansion of Th17 and Th22 populations that secrete IL-6, IL-17, and IL-22, key mediators of epidermal hyperplasia and chronic inflammation [70,72].

In addition to adaptive immunity, innate immune components, including dendritic cells, macrophages, neutrophils, natural killer (NK) cells, and even keratinocytes, play a significant role in amplifying the inflammatory cascade via the release of pro-inflammatory cytokines [70]. In psoriasis, self-DNA and LL-37 can activate plasmacytoid dendritic cells, leading to type I interferon release and stimulation of Th1/Th17 cells. This inflammatory environment promotes keratinocyte hyperproliferation, leukocyte infiltration, and neoangiogenesis in affected skin areas [70].

Vitamin D, particularly in its active form, 1,25(OH)2D, plays a multifaceted role in the pathogenesis and potential treatment of psoriasis, exerting effects on both the immune system and epidermal homeostasis [18]. At the innate immune level, vitamin D enhances the function of dendritic cells, macrophages, and T lymphocytes by boosting their phagocytic activity and stimulating AMP production, notably cathelicidin. These AMPs reinforce the skin’s defense against microbial colonization, which is frequently disrupted in psoriatic plaques [73]. Specifically, plasmacytoid dendritic cells (pDCs), key initiators of psoriatic inflammation, express both VDR and vitamin D-metabolizing enzymes. Vitamin D signaling in these cells suppresses their activation, limiting T-cell stimulation and IFN-γ secretion [74]. In the adaptive immune system, vitamin D promotes immune balance by inhibiting Th1 cell proliferation and their associated cytokines IL-2 and IFN-γ, while simultaneously encouraging a Th2 cytokine profile, favoring IL-4 and IL-10 expression, which helps dampen chronic inflammation [75]. It also downregulates the Th17 axis, reducing skin infiltration and activity of Th17 cells that are responsible for producing IL-17, IL-22, and other psoriasis-related cytokines [76,77]. Furthermore, vitamin D suppresses pro-inflammatory mediators elevated in psoriatic lesions, including IL-1α, IL-1β, Tumor Necrosis Factor (TNF)-α, and the IL-12/23p40 subunit [78,79].

Beyond cytokine regulation, vitamin D modulates downstream inflammatory proteins central to psoriatic pathology. For instance, treatment with calcipotriol (a vitamin D analog) reduces the expression of Th17-induced molecules such as psoriasin (S100A7) and koebnerisin (S100A15), which act as chemoattractants and amplify skin inflammation [80]. In reconstructed psoriatic skin models, the analog 1α,25(OH)_2_D_3_–3-bromoacetate exhibited stronger antiproliferative activity than calcitriol, reversing IL-22–induced signaling pathways and downregulating AKT1, mTOR, IL-8, RANTES, and psoriasin [81].

Vitamin D also contributes to skin barrier integrity and keratinocyte homeostasis. Through VDR binding in keratinocytes, it promotes the expression of barrier proteins such as filaggrin, loricrin, and involucrin, components frequently deficient in psoriatic skin [70,82]. Hosomi et al. showed that vitamin D suppresses DNA synthesis in keratinocytes, promoting terminal differentiation and the formation of cornified envelopes, effectively counteracting keratinocyte hyperproliferation typical of psoriatic lesions [83].

Additionally, vitamin D influences TJ protein expression, with VDR activity being linked to the regulation of claudin, ZO-1, and occluding, molecules that are often downregulated in psoriatic epidermis [84]. It also modulates integrins and immune activation markers such as ICAM-1, CD26, and HLA-DR, contributing to normalization of skin structure and immune surveillance [85].

Emerging research has also explored VDR gene polymorphisms as genetic contributors to psoriasis susceptibility. Variants such as A-1012G, FokI, BsmI, ApaI, and TaqI have shown associations with disease risk in Italian and Chinese cohorts, while no significant associations were found in studies from Croatia and Egypt—suggesting that genetic effects may be population-specific [86,87,88,89,90]. Furthermore, these polymorphisms may predict treatment response: the TaqI variant has been linked to shorter remission following NB-UVB phototherapy, and wild-type alleles of A1012G, FokI, and TaqI have correlated with improved outcomes after calcipotriol treatment [91,92,93].

Taken together, these findings emphasize the multifactorial role of vitamin D in psoriasis, from immunoregulation and epidermal repair to potential pharmacogenetic implications, and support its use as both a therapeutic agent and biomarker in managing the disease.

### 2.4. Clinical and Epidemiological Evidence Linking Vitamin D to Psoriasis

A substantial body of clinical and epidemiological research has investigated the association between vitamin D status and psoriasis, with many studies reporting significantly reduced serum 25(OH)D levels in psoriatic patients compared to healthy controls [54,57,94,95,96]. For example, Chandrashekar et al. [55] and Maleki et al. [54] observed markedly lower serum vitamin D levels in individuals with psoriasis, with an inverse correlation to PASI scores, suggesting that more severe disease is often associated with more profound vitamin D deficiency [54,55]. Similar findings were confirmed by Bergler-Czop et al. [95] and Pokharel et al. [97], reinforcing the link between hypovitaminosis D and disease burden. However, Wilson et al. [98] reported contradictory data in a large population-based screening, finding no significant difference in serum vitamin D levels between patients with or without psoriasis. These discrepancies may be attributed to confounding variables such as sun exposure, dietary intake, seasonality, race, and baseline vitamin D status, underscoring the need for cautious interpretation.

Several clinical trials have assessed the therapeutic impact of vitamin D supplementation. Finamor et al. [56] demonstrated that oral administration of 35,000 IU/day of vitamin D_3_ for six months led to significant PASI improvement, alongside a robust rise in serum 25(OH)D levels [56]. Conversely, trials using high-dose monthly supplementation (e.g., 100,000 IU) failed to achieve consistent clinical benefits despite biochemical normalization, as seen in studies by Ingram and Jarrett [57,58]. Additionally, Prystowsky et al. [99] found no synergistic benefit when combining oral calcitriol with NB-UVB phototherapy [99], while Gumowski-Sunek et al. [100] reported disturbances in calcium metabolism with oral forms not observed in topical applications [100]. However, topical vitamin D analogs, such as calcipotriol, tacalcitol, and maxacalcitol, remain first-line options in mild to moderate psoriasis due to their favorable efficacy and safety profiles [101]. Calcipotriol has shown clinical superiority when combined with NB-UVB or corticosteroids and is capable of selectively reducing IL-6 expression in psoriatic lesions [102,103,104,105]. Tacalcitol and maxacalcitol also demonstrate effective PASI reduction without altering calcium homeostasis, although the latter carries a slightly higher hypercalcemia risk at systemic doses [106,107,108].

Notably, the combination of vitamin D analogs with corticosteroids has proven more effective than monotherapy, offering complementary mechanisms. In this regard, vitamin D restores epidermal differentiation, while corticosteroids mitigate inflammation and local irritation [109,110,111]. Furthermore, NB-UVB and UVA/UVB phototherapy are believed to exert part of their therapeutic efficacy via increased endogenous vitamin D synthesis [107,112].

Despite these promising findings, variability among studies remains, largely due to differences in dosage, duration, formulations, baseline vitamin D status, and patient heterogeneity. This limits definitive conclusions regarding the optimal use of systemic supplementation. Moreover, most clinical trials focus on established moderate-to-severe psoriasis, leaving open the question of whether vitamin D could play a preventive or disease-modifying role in early or subclinical stages of psoriasis (Table 2).

Taken together, the evidence supports vitamin D as a clinically relevant modulator of epidermal differentiation and inflammation in psoriasis, with potential application in therapeutic strategies pending more standardized supplementation trials.

## 3. Vitamin D and Cardiovascular Health

Psoriasis is not only a chronic inflammatory skin condition, but also a systemic disorder increasingly recognized for its association with cardiovascular comorbidities. Patients with psoriasis exhibit a significantly higher risk of developing cardiovascular disease (CVD), including myocardial infarction, stroke, and metabolic syndrome. This relationship is thought to be mediated by shared inflammatory pathways and altered vitamin D metabolism [113]. These findings underline the broader systemic implications of vitamin D dysregulation in psoriatic patients. Transitioning from its dermatologic roles, vitamin D is also a crucial modulator of cardiovascular health. The presence of VDRs in cardiomyocytes, endothelial and vascular smooth muscle cells (VSMCs), fibroblasts, and pericytes indicates its capacity to influence blood pressure regulation and attenuate inflammation within the cardiovascular system [114]. This intersection highlights the relevance of vitamin D not only in skin homeostasis, but also in maintaining vascular integrity and mitigating cardiovascular risk (Figure 1; Table 1).

Furthermore, considering that TJs are present in all epithelia, not only in the epidermis, and based on the observation that patients with psoriasis more frequently present with IBD, a study [115] was conducted on the serum of patients with psoriasis to assess increased epithelial permeability, particularly of the intestinal epithelium. The study revealed elevated levels of both zonulin and lipopolysaccharide (LPS). As is well known, LPS is a component of the outer membrane of Gram-negative bacteria and plays a key role in the pathogenesis of atherosclerosis [116].

### 3.1. Pathogenesis of Cardiovascular Disease and Molecular Actions of Vitamin D

CVDs are multifactorial conditions driven by endothelial dysfunction, vascular inflammation, oxidative stress, dysregulation of the RAAS, lipid abnormalities, and impaired glucose metabolism [117].

Vitamin D plays a crucial role in maintaining endothelial homeostasis, essential for vascular health and atherosclerosis prevention, by reducing oxidative stress, lowering reactive oxygen species (ROS), and enhancing nitric oxide (NO) bioavailability [118]. In vitro experiments using human umbilical vein endothelial cells (HUVECs) show that calcitriol downregulates leptin-induced inflammation by suppressing VCAM-1 and MCP-1 expression and inhibiting the NF-κB pathway, while concurrently promoting NO production [119,120,121]. In a controlled clinical trial, calcitriol and cholecalciferol were randomly assigned to patients undergoing elective PCI, demonstrating a lowering effect of calcitriol on hs-CRP serum levels [122]. Additionally, vitamin D enhances angiogenesis by activating endothelial colony-forming cells (ECFCs), which are key players in vascular regeneration [123].

Vitamin D also exerts antioxidant activity via two key transcriptional regulators. The first is nuclear factor erythroid 2–related factor 2 (Nrf2), which induces the transcription of antioxidant enzymes such as catalase (CAT), γ-glutamyltransferase (γ-GT), glucose-6-phosphate dehydrogenase (G6PD), glutathione peroxidases (Gpx), glutathione (GSH), thioredoxin reductase (TR), glutathione reductase (GR), superoxide dismutase 1/2 (SOD1/2), and thioredoxin (TRX) [124]. The second is Klotho, which also enhances antioxidant capacity by upregulating CAT, peroxiredoxins 2/3 (Prx-2/3), SOD2, and Trxrd-1, and contributes to calcium regulation [125,126].

Vitamin D modulates intracellular calcium signaling through proteins such as plasma membrane Ca^2+^-ATPase (PMCA), calbindin, Na^+^/Ca^2+^ exchanger (NCX1), parvalbumin, and transient receptor potential V members 5 and 6 (TRPV5/6), which influence vascular tone, cardiac contractility, and insulin secretion [127].

In VSMCs, 1,25(OH)2D3 suppresses the secretion of IL-6 and TNF-α, inhibits proliferation, prevents osteogenic transdifferentiation, and promotes prostacyclin synthesis [128,129]. VSMCs derived from VDR-knockout mice show increased expression of angiotensin II type 1 receptors, confirming vitamin D’s role in vascular tone regulation [130].

Hypertension is a well-established risk factor for CVD, and vitamin D deficiency has been strongly associated with its pathogenesis [131]. The RAAS is a hormone system that regulates blood pressure and fluid balance through renin, angiotensin II, and aldosterone [132]. Vitamin D inhibits renin expression, thereby downregulating the RAAS cascade and decreasing both angiotensin II and aldosterone levels. These effects ultimately lower blood pressure and reduce hypertensive damage [133]. VDR-null mice demonstrate elevated RAAS activity, increased sympathetic tone, and raised intraglomerular pressure, which together promote hypertension [134]. However, large RCTs (VitDISH, Styrian Trial) demonstrated no clinically significant effects of vitamin D supplementation [135,136]. Only the Iranian trial showed modest blood pressure reductions but its duration and sample limited applicability [137].

Vitamin D helps prevent atherogenesis by favorably modulating lipid profiles, reducing total cholesterol, LDL, and triglycerides, while increasing HDL levels [138]. It induces the expression of ATP-binding cassette transporter A1 (ABCA1), which promotes cholesterol efflux from macrophages in atherosclerotic plaques [139] and regulates lipid-metabolizing enzymes such as lipoprotein lipase and hepatic lipase, which are essential for maintaining optimal HDL and LDL levels [140]. Moreover, vitamin D deficiency contributes to LDL accumulation, oxidation, and foam cell formation, hallmarks of early atherogenesis [127]. Indeed, it has been reported that vitamin D supplementation might exert anti-inflammatory effects, by acting on IL-6 levels, VCAM and E-selectin [141]. In a meta-analysis including 3 RCTs no significant association was found between serum vitamin D levels and prevention or regression of atherogenesis, as measured by cIMT (carotid intima-media thickness) [142].

Beyond lipid metabolism, vitamin D influences glucose homeostasis and insulin responsiveness, both key to cardiovascular health. Insulin resistance (IR) contributes to endothelial dysfunction, inflammation, and accelerated atherogenesis [143]. Pancreatic β cells express both VDR and the 1α-hydroxylase enzyme (Cyp27b1), allowing local synthesis of active vitamin D [140,144]. Animal models show that vitamin D deficiency impairs insulin secretion, which is reversible upon supplementation [145,146]. These effects are mediated through VDR-dependent gene induction [147,148] and calcium-driven insulin exocytosis involving PKA signaling and endoplasmic reticulum calcium mobilization [149,150]. Vitamin D may induce improvements in insulin resistance, as measured by glycemic markers (HbA1c, fasting glucose, HOMA-IR), though effect sizes are small and incidence of diabetes remained unaffected [151,152].

Vitamin D also protects cardiomyocytes from hyperglycemia-induced stress and angiotensin II–mediated hypertrophy [114], promotes cardiomyoblast survival and proliferation [153], and modulates immune responses by decreasing pro-inflammatory cytokines (TNF-α, IL-1β, IL-6) while enhancing IL-10 production and M2 macrophage polarization [154]. Animal models with targeted deletion of VDR or 1α-hydroxylase genes demonstrate increased RAAS activity, myocardial hypertrophy, fibrosis, apoptosis, and systolic dysfunction, emphasizing the essential role of vitamin D signaling in cardiac structure and function [127]. The effect of vitamin D supplementation on left ventricular mass has been explored in CKD, hypertensive or general older adults, showing no significant regression, as showed by using cardiac magnetic resonance (36008108), except in HF populations [155,156].

Collectively, these findings suggest that vitamin D influences multiple pathogenic pathways in cardiovascular disease, acting on endothelial function, oxidative stress, lipid and glucose metabolism, and vascular inflammation, while clinical data highlight both its therapeutic promise and the need for more definitive intervention trials.

### 3.2. Clinical Implications of Vitamin D Deficiency in Cardiovascular Disease: Conditions and Evidence

Vitamin D deficiency has been consistently associated with several clinical conditions that predispose to CVD, including IR, metabolic syndrome (MetS), acute coronary syndromes (ACS), and heart failure (HF) [11,61]. These disorders share key pathogenic mechanisms such as chronic inflammation, oxidative stress, endothelial dysfunction, and calcium metabolism dysregulation [11,61].

Observational studies report an inverse association between serum vitamin D levels and the Homeostatic Model Assessment of Insulin Resistance (HOMA-IR), a validated marker of IR based on fasting glucose and insulin levels [157]. Vitamin D also plays a critical role in β-cell function and skeletal muscle glucose uptake, influencing whole-body energy homeostasis and insulin sensitivity [158,159,160]. Considering that skeletal muscle accounts for up to 90% of postprandial glucose disposal, this relationship has clinical relevance.

Vitamin D modulates adipose tissue metabolism, downregulates pro-inflammatory cytokines, and exerts protective anti-inflammatory effects on pancreatic β-cells. In obesity, a common condition in IR and T2DM, vitamin D sequestration in adipose tissue reduces its bioavailability, contributing to a cycle of metabolic dysfunction [11]. A growing body of evidence links low 25(OH)D levels to MetS, characterized by central obesity, IR, hypertension, and dyslipidemia. Observational studies suggest that every 10 ng/mL increase in vitamin D levels may reduce the risk of MetS by 15–20% [161,162]. Indeed, in a meta-analyses involving >80,000 individuals suggest that benefits may be confined to subgroups such as the elderly, individuals with MetS, or those with severe baseline deficiency [163,164].

The cross-sectional InCHIANTI study revealed an inverse association between both 25(OH)D and 1,25(OH)2D and cardiovascular risk in 299 elderly individuals. Notably, over 60% of participants had serum levels <20 ng/mL [12]. Both serum levels of 25(OH)D and 1,25(OH)2D were negatively correlated with cardiovascular risk, as measured by the SCORE2/SCORE2-OP algorithm, a 10-year CV risk estimator endorsed by the European Society of Cardiology [12].

Interventional studies suggest that vitamin D supplementation improves surrogate markers of glucose metabolism. Significant improvements in HOMA-IR and HbA1c have been reported, particularly among insulin-resistant or obese individuals [165,166]. Randomized trials and meta-analyses also show beneficial effects on insulin sensitivity, β-cell function, and fasting glucose levels in high-risk groups [167,168,169,170,171,172]. Additionally, favorable outcomes on HDL-C and C-reactive protein (CRP) have been noted [170].

However, findings remain inconsistent. Several trials failed to show significant changes in anthropometric or lipid parameters, particularly in individuals without baseline vitamin D deficiency [173,174,175].

Vitamin D supplementation has also been linked to reductions in systolic blood pressure, possibly mediated by suppression of the RAAS and secondary hyperparathyroidism [11]. These mechanisms may offer protection against hypertension and vascular stiffness.

In individuals with type 2 diabetes mellitus T2DM, a condition marked by hyperglycemia and impaired insulin secretion, vitamin D deficiency is associated with increased risk of both microvascular and macrovascular complications [176]. Epidemiological studies link low vitamin D to diabetic retinopathy, nephropathy, neuropathy, and foot ulcers [177,178,179]. Mechanistically, vitamin D modulates nociceptor activity in neuropathy [90], regulates angiogenesis and inflammation in retinopathy [179], and reduces proteinuria in diabetic nephropathy [11].

In the macrovascular context, vitamin D deficiency is associated with endothelial dysfunction, arterial stiffness, peripheral artery disease (PAD), and carotid plaque formation [180,181,182]. The Framingham Offspring Study showed increased risk of myocardial infarction in individuals with serum 25(OH)D levels <15 ng/mL [183].

In HF patients, low vitamin D is linked to elevated BNP, cardiac remodeling, and reduced ejection fraction. Animal studies suggest a causal relationship with myocardial hypertrophy and fibrosis, although clinical trials such as VITAL-HF failed to demonstrate a reduction in hard endpoints like hospitalization or mortality [163,184]. However, a meta-analysis, considering seven RCTs (465 patients undergoing to vitamin D supplementation or placebo over 12 weeks to 9 months), reported a significant reduction in LV end-diastolic diameter (−2.31 mm) and an improvement of LVEF (+4.18%) [185].

Genetic predisposition may influence individual responses to vitamin D and explain heterogeneity across studies. A meta-analysis involving 9232 individuals revealed associations between specific VDR polymorphisms (ApaI, BsmI, TaqI) and IR-related disorders, with ethnic differences in susceptibility. For instance, the ApaI G allele was linked to MetS and PCOS in Asian and mid-latitude populations, whereas FokI showed no significant associations [163].

In HF, VDR variants may affect myocardial fibrosis and remodeling, further complicating the interpretation of vitamin D trial results [186,187]. Additionally, vitamin D deficiency promotes secondary hyperparathyroidism, leading to intracellular calcium accumulation, systemic inflammation, and worsening IR [188,189].

Despite robust observational evidence linking low vitamin D to a wide range of cardiovascular and metabolic outcomes [127], RCTs and meta-analyses have yielded mixed results.

Large primary prevention RCTs such as ViDA [190], VITAL [60], and D-Health [191] have generally reported null effects of vitamin D supplementation. A major concern is the lack of stratification by baseline status; in VITAL’s ancillary fracture analysis (LeBoff et al.), 87 percent entered with 25(OH)D above the 50 nmol/L (>20 ng/mL) threshold, limiting the chance to detect benefit [192]. While RCTs evaluating the effects of vitamin D supplementation on cardiovascular risk do offer valuable causal insights, they are often limited by relatively small sample sizes, short follow-up duration, narrow inclusion/exclusion criteria, and limited generalizability.

Another fundamental weakness of these trials relates to heterogeneity of the populations, comorbidities, and endpoints [114]. Age, gender, and physical activity influence absorption, metabolism, and responsiveness, while diabetes, obesity, hypertension, and frailty further modify outcomes. Interactions with diet, medications, and BMI complicate interpretation by altering bioavailability. Older adults, women, and deficient individuals may see greater benefit, yet inconsistent trial designs and diverse endpoints contribute to conflicting evidence, highlighting the need for personalized approaches. These and other limitations of trials to evaluate vitamin D benefit have been discussed in detail elsewhere [13,114,193,194].

Nonlinear Mendelian randomization (NMR) studies complement RCTs by evaluating long-term vitamin D exposure, deficiency, and dose–response effects on cardiovascular outcomes in real-world settings. Recent evidence from a large NMR study by Sutherland and colleagues shows mortality risk rises steeply below 50 nmol/L with a plateau at higher levels [195], implying little benefit in replete cohorts and helping explain null trial results. Supporting this hypothesis, Patriota et al. reported a significant inverse association between 25(OH)D and cardiovascular events over 14.4 years, especially in continuous analyses, with the largest effects observed at concentrations above 50 nmol/L [196].

Taken together, null RCTs likely reflect enrollment of vitamin D replete populations and design limits rather than a lack of effect. NMR and cohorts indicate benefit mainly below the threshold of 50 nmol/L. Future trials should stratify by baseline status and enrich for deficiency. These insights are summarized in Table 2. The findings also support the interpretative model proposed by Tripepi et al. [13], who argue that intervention is unlikely to yield benefit above the threshold of 20 ng/mL. Their analysis, based on NMR, identified a sharp rise in mortality below this level, thus reinforcing 20 ng/mL as a critical biological and prognostic cut-off [13]. These discrepancies highlight the need for stratified clinical trials and personalized intervention strategies.

Overall, current evidence links vitamin D deficiency to insulin resistance, metabolic syndrome, acute coronary syndromes, and heart failure, with observational data consistently supporting increased risk, while results from randomized trials highlight the need for stratified designs that target deficient populations in order to clarify true cardiovascular benefit.

## 4. Vitamin D and Intestinal Bowel Disease

Chronic inflammation and compromised epithelial barriers are key features in IBD, particularly CD and UC, and in celiac disease (CeD) [197]. These conditions involve disruption of TJs between enterocytes, leading to increased intestinal permeability [198]. This “leaky gut” permits the translocation of microbial products and dietary antigens into the lamina propria, fueling mucosal immune activation and sustaining a vicious cycle of inflammation and barrier breakdown [199]. In IBD, barrier dysfunction often precedes relapse and correlates with disease severity [200]; in CeD, adaptive zonulin release triggered by gluten peptides (via the CXCR3/MyD88 pathway) also disrupts TJ integrity, facilitating antigen crossing and autoimmune activation [201] (Figure 1; Table 1).

### 4.1. Modulation of Gut Barrier Function by Vitamin D: Epithelial, Immune, and Microbial Interactions

The intestinal barrier, comprising microbiota, mucus, epithelium, and immune cells, maintains gut and systemic homeostasis [202]. Its disruption can trigger multifactorial diseases [203]. Microbial-immune crosstalk is mediated by specialized gut structures [204,205], while the microbiota supports pathogen defense and nutrient metabolism [206,207]. The mucus layer and epithelial cells ensure selective permeability and immune protection [208,209], with immune cells in the lamina propria orchestrating surveillance and signaling [210].

Vitamin D contributes to intestinal barrier integrity through its actions on epithelial cells, immune responses, and host–microbiota interactions [211,212]. VDR signaling plays a central role in maintaining TJ integrity, as shown in models of IBD and CeD [213,214,215,216]. In VDR-deficient mice, claudin-1 and -3 expression is reduced, junctions appear disorganized, and permeability increases. VDR agonists can restore TJ protein levels and epithelial structure after inflammation [217]. Calcitriol also prevents gliadin-induced barrier disruption in CeD by inhibiting zonulin-mediated TJ disassembly [218], while cholecalciferol restores villus morphology and ZO-1 organization in gluten-sensitive mice [8].

Beyond structural effects, vitamin D also prevents epithelial cell death, strengthens adhesion, and improves resilience to microbial and toxic challenges [219]. It also modulates mucosal immunity by promoting regulatory T cell differentiation and suppressing Th1/Th17 responses [220,221].

In addition, vitamin D indirectly regulates the gut microbiota via epithelial and immune cell VDR signaling [212]. VDR deficiency leads to dysbiosis, impaired Paneth cell function, defective antimicrobial peptide secretion (e.g., lysozyme, defensin-4), and increased inflammation in mouse models [222,223,224]. On the other hand, supplementation with 1,25(OH)_2_D partially reverses dysbiosis [225], and higher vitamin D levels correlate with increased cathelicidin and reduced inflammation in UC patients [226].

Vitamin D also promotes epithelial regeneration. VDR-deficient mice show impaired wound healing and crypt repair in colitis models. Regenerative effects have been observed in CeD mouse models treated with high-dose vitamin D, preserving villus/crypt architecture and reducing lymphocyte infiltration [8].

In particular, oral cholecalciferol at increasing doses (5–130 µg/kg) was administered to gluten-sensitive Non-Obese Diabetic (NOD/ShiLtJ) mice fed a gluten-containing diet and receiving gliadin to induce enteropathy [8]. Compared to untreated CeD controls (gliadin + vehicle), vitamin D significantly reduced mucosal lesion severity and increased villus length in a dose-dependent manner. Only high-dose groups (50–130 µg/kg) showed near-complete restoration of villus/crypt architecture. Immunohistochemistry confirmed reduced CD3+ lymphocyte infiltration and ZO-1 expression, indicating improved epithelial integrity and barrier function [8].

Collectively, these findings support a multifaceted role of vitamin D in maintaining gut barrier function. Through enhancement of epithelial cohesion, modulation of immunity, support of microbial homeostasis, and promotion of regeneration, vitamin D/VDR signaling helps counteract the cycle of barrier dysfunction and chronic inflammation characteristic of IBD and CeD [212].

### 4.2. Vitamin D and Inflammatory Bowel Disease and Celiac Disease: Evidence from Clinical Trials and Meta-Analyses

Patients with IBD, have a 64% greater likelihood of vitamin D deficiency [227], with prevalence reaching 38.1% in CD and 31.6% in UC [228]. Deficiency is linked to increased disease activity, poor quality of life, and risk of relapse [62].

In particular, vitamin D deficiency is frequently observed in CeD, with pediatric prevalence ranging from 9 to 52% [229]. A meta-analysis of 24 studies (1137 CeD patients, 2613 controls) found mean serum 25(OH)D levels were 3.34 ng/mL lower in CeD patients, supporting impaired absorption due to intestinal inflammation [230]. While no human trials have tested vitamin D’s direct effect on CeD pathology, animal models show that high-dose cholecalciferol ameliorates villus atrophy, reduces CD3+ infiltration, and restores ZO-1 expression [8].

Vitamin D supplementation has demonstrated immunomodulatory benefits in both pediatric and adult IBD populations. Systematic reviews report reductions in CRP and ESR, and early signs of clinical improvement, despite suboptimal target attainment [66]. RCTs in adults confirm reduced disease activity, fecal calprotectin, and CRP at weekly doses of 2000–50,000 IU, with no major adverse events [229,231,232]. Meta-analyses show decreased relapse rates (RR 0.64), particularly in CD patients in remission (RR 0.47) [233], although effects on ESR and symptom scores remain inconsistent [234].

Microbiome studies further support vitamin D’s role in gut homeostasis. Supplementation increases microbial richness and beneficial taxa [235,236], although results vary by dose and context [237,238]. Certain microbes modulate VDR expression and vitamin D metabolism (e.g., via FGF-23, CYP27B1), and produce bioactive metabolites such as lithocholic acid, enhancing absorption [211,239]. Lactobacillus reuteri supplementation raised 25(OH)D_3_ levels in humans [239], while butyrate and Bifidobacteria have been positively linked to vitamin D levels [238].

Together, these findings highlight the emerging role of vitamin D as a modifiable factor in CeD and IBD management. Beyond correcting deficiency, repletion of vitamin D alongside a gluten free diet or standard therapies contributes to mucosal integrity, reduced inflammation, improvement in quality of life, and potentially enhancing pharmacological response (Table 2). The routine screening and targeted supplementation of specific patients are warranted.

## 5. Vitamin D and Epithelial Integrity in Chronic Respiratory Disease

Vitamin D also exerts important effects on epithelial barrier integrity and immune regulation in the respiratory system, which were not the main focus of the present review and have been discussed in detail elsewhere [240,241]. Deletion of the VDR has been shown to disrupt TJs in the lungs, leading to impaired barrier function and increased susceptibility to inflammatory damage, thereby implicating VDR signaling as a key determinant of pulmonary epithelial integrity in conditions such as pneumonia, asthma, and chronic obstructive pulmonary disease (COPD) [242].

In some specific viral infections, including COVID-19, vitamin D deficiency has been associated with dysregulated immune responses and heightened disease severity [9,240,241,243]. Mechanistic insights highlight the capacity of vitamin D to modulate innate and adaptive immunity, attenuate cytokine storm pathways, and enhance epithelial resilience [243,244]. The use of vitamin D supplementation, particularly at high doses has shown important benefit in patients with COVID-19 [9,245,246,247,248].

In addition, there is tentative evidence linking vitamin D to cancer [249,250], where it may exert antiproliferative and immunomodulatory effects [251]. For example, VDR expression in melanocytes and melanoma cells suggests a direct influence on tumor growth and immune evasion, and there is evidence from some epidemiological studies linking vitamin D deficiency to worse cancer prognosis [14,252,253].

These findings extend the role of vitamin D beyond the skin, gut, and cardiovascular system, supporting its systemic relevance in chronic inflammatory diseases characterized by barrier dysfunction.

Collectively, these data emphasize that maintaining sufficient vitamin D levels is critical for epithelial barrier protection and immune homeostasis across diverse organ systems and chronic diseases.

## 6. Safety of Vitamin D Supplementation

In general, there is still a lack of consensus on the recommended vitamin D supplementation regimen (doses, administration schedule, duration of treatment) [254]. This heterogeneity can be explained in part by the lack of pharmacokinetic studies evaluating different dosing schedules [255,256,257]. Safer strategies include moderate daily supplementation (800–1000 IU cholecalciferol or 10 µg calcifediol) [5,258,259,260,261,262,263].

Daily dosing is generally safer than intermittent high/megadoses, which have been linked to increased falls and fractures [264,265]. The National Academy of Medicine sets the upper tolerable intake at 4000 IU/day, although some evidence suggests risk may occur below this.

Excessive vitamin D can worsen granulomatous conditions such as sarcoidosis, where macrophage-driven calcitriol leads to hypercalcemia [266]. In addition, high-doses of vitamin D have also been associated with increased risk of hypercalciuria and kidney stones, particularly when combined with calcium [267].

In heart failure, supplementation with 4000 IU/day for 3 years did not reduce mortality [268]. Gastrointestinal implications include the use of calcifediol, which has superior absorption in liver disease and gastrointestinal malabsorption [269]. While doses up to 10,000 IU/day have been tested without acute toxicity, caution is warranted. Large boluses (≥300,000 IU intramuscularly) are discouraged due to adverse outcomes; safer intermittent boluses should not exceed 100,000 IU [260,270].

## 7. Conclusions

Vitamin D plays an essential role in maintaining the structural and immunological integrity of epithelial barriers, regulating innate and adaptive immune responses, and contributing to cardiovascular and gastrointestinal health. Its actions extend far beyond traditional skeletal functions, influencing pathophysiological processes in atopic dermatitis, psoriasis, metabolic syndrome, inflammatory bowel disease, and CeD. While observational and mechanistic studies provide strong support for these effects, results from supplementation trials remain variable, often depending on baseline deficiency, genetic factors, and disease state.

The cumulative evidence suggests that maintaining adequate vitamin D levels is a promising adjunctive strategy for managing chronic inflammatory conditions. However, more well-designed, stratified clinical trials are needed to clarify optimal dosing, target populations, and biomarkers of response. Future research should focus on integrating vitamin D status into personalized care approaches and elucidating its crosstalk with microbiota, epithelial regeneration pathways, and immune checkpoints in disease modulation.

## 8. Literature Search

In the preparation of this review, we performed a comprehensive literature search to identify relevant studies on the non-skeletal role of vitamin D. PubMed/Medline (until June 2025) was searched using the following keywords: (“vitamin D” OR cholecalciferol OR ergocalciferol OR “25-hydroxyvitamin D” OR 25OHD) AND (psoriasis OR “atopic dermatitis” or eczema OR IBD OR Crohn’s OR Celiac OR “ulcerative colitis” OR cardiovascular OR CVD OR stroke OR “acute myocardial infarction” OR diabetes or “metabolic syndrome”). We repeated this search using different Boolean variations and also with additional keywords focused on “non-skeletal” or extra-skeletal”. We also performed an additional search to add evidence on the role of vitamin D on TJ in respiratory diseases such as asthma, COVID-19 and chronic obstructive pulmonary disease, or COPD. We have included original research articles, clinical trials, meta-analyses, and high-quality reviews published in the previous 15 years. Studies that were not published in English language in addition to hand-selected case studies, abstracts, letters, and reviews were excluded. Articles not related or not relevant to the non-skeletal role of vitamin D or the topic discussed were also omitted.

## Figures and Tables

**Table 1:** Extra-skeletal effects of vitamin D on skin, gut, and cardiovascular system, highlighting key molecular targets and clinical evidence from epidemiological and interventional studies.

Organ/System	Effects of Vitamin D	Key Markers/Proteins	Main Clinical/Epidemiological Evidence
Skin (AD, Psoriasis)	Strengthens epidermal barrier via TJs; promotes keratinocyte differentiation; boosts antimicrobial peptides; modulates immune response	Claudin-1, Occludin, ZO-1, Filaggrin, Cathelicidin, VDR, CYP27B1, CYP24A1	Lower severity in AD/PSO patients; supplementation (1500–1600 IU/day) improves SCORAD/PASI; beneficial in genetic variants
Intestine (IBD, CeD)	Maintains epithelial barrier; regulates TJs; modulates immunity and microbiota; reduces epithelial apoptosis; promotes Notch-1–mediated regeneration	Claudin-1/2/3/15, Occludin, ZO-1, VDR, Cathelicidin, CD3+ T cells, Notch-1	Supplementation reduces disease activity, relapse; improves mucosal healing and microbiota; better response in patients with <30 ng/mL
Cardiovascular system (CVD)	Reduces inflammation and oxidative stress; regulates RAAS and endothelial function; modulates lipid/glucose metabolism; protects cardiomyocytes	NO, VCAM-1, MCP-1, Nrf2, Klotho, PMCA, TRPV5/6, ABCA1, SOD, IL-6, TNF-α	Observational studies show lower CVD risk with higher vitamin D; interventional data mixed

AD, Atopic Dermatitis; PSO, Psoriasis; TJ, Tight Junctions; IBD, Inflammatory Bowel Disease; CeD, Celiac Disease; VDR, Vitamin D Receptor; NO, Nitric Oxide; ZO-1, Zonula Occludens-1; RAAS, Renin–Angiotensin–Aldosterone System; VCAM-1, Vascular Cell Adhesion Molecule 1; MCP-1, Monocyte Chemoattractant Protein 1; Nrf2, Nuclear Factor Erythroid 2–Related Factor 2; PMCA, Plasma Membrane Ca^2+^ ATPase; TRPV5/6, Transient Receptor Potential Vanilloid 5/6; CVD, Cardiovascular Disease.

**Table 2:** Key clinical and interventional evidence on the non-skeletal effects of vitamin D. This table summarizes the most relevant observational and interventional studies supporting the non-skeletal effects of vitamin D in atopic dermatitis, psoriasis, IBD, celiac disease, and cardiovascular disease.

Disease/Condition	Study Design [Reference]	Subjects/Sample Size	Primary Outcome/Endpoints	Main Findings on Vitamin D
Atopic Dermatitis (AD)	Cross-sectional [37]	681 children/young adults	Serum 25(OH)D vs. AD severity (EASI)	84% of AD patients were vitamin D deficient; levels inversely correlated with disease severity
	Meta-analysis [40,41]	11 RCTs, *n* = 686	SCORAD, EASI scores	Supplementation (1500–1600 IU/day ≥ 12 wks) significantly reduced severity
	RCT [53]	107 children	AD severity after vitamin D or placebo	Supplementation improved EASI vs. placebo
	Observational [36]	106 children	Serum 25(OH)D and AD severity	Lower 25(OH)D in moderate-severe AD; negative correlation
Psoriasis (PSO)	Cross-sectional [54,55]	100–300	Serum 25(OH)D vs. PASI	Lower vitamin D in psoriasis; inverse correlation with PASI
	RCT [56]	25 patients	Oral vitamin D3 (35,000 IU/d, 6mo), PASI	Significant improvement in PASI, ↑25(OH)D
	RCT [57,58]	>200 total	High-dose vitamin D3 (monthly/weekly)	No significant clinical improvement vs. placebo
	Meta-analysis [59]	8 studies, *n* = 4349	Serum 25(OH)D and psoriasis risk	Low vitamin D associated with increased risk
Cardiovascular Disease (CVD)	Meta-analysis [60]	>80,000 (multiple studies)	CVD risk, MI, stroke, MACE, BP	Low vit D = ↑CVD risk (observational studies); supplementation effect on MACE inconsistent, some benefit in subgroups
	Cohort Study [12]	>1000 elderly	25(OH)D/1,25(OH)2D vs. CVD risk	Low vit D linked to higher CVD risk, obesity, inflammation
	Meta-analysis [11,61]	>17 RCTs, >8000 subjects	Lipids, BP, HOMA-IR, CRP	Mixed results: some ↓CRP, fasting glucose, HOMA-IR; limited effect on major CVD outcomes
IBD (CD + UC)	Meta-analysis [62]	8316 IBD patients	Disease activity, relapse, QOL	Low vitamin D = ↑activity, relapse, worse QOL
	Observational [63]	470 IBD patients	Serum 25(OH)D and disease activity	25(OH)D inversely correlated with disease activity
	RCT [64]	94 CD patients	Vit D3 (1200 IU/d) vs. placebo, relapse	Supplementation reduced relapse risk
	Cross-sectional [65]	94 CD	25(OH)D levels, CDAI	25(OH)D levels correlated with CDAI
	Meta-analysis [66]	10 trials, pediatric IBD	25(OH)D, CRP, ESR, activity	Safe supplementation; ↓CRP/ESR, trend to benefit
	Meta-analysis [67]	27 studies, *n* = 8316	Disease activity, relapse, QOL	Low vit D: ↑activity (OR 1.53), relapse (OR 1.23), poor QOL
Celiac Disease (CeD)	Meta-analysis [62]	24 studies; 1137 CeD; 2613 ctrl	Serum 25(OH)D, effect GFD	Mean 25(OH)D lower by 3.3-fold ng/mL; improved with GFD
	Prospective [68]	33 pediatric CeD	Vitamin D (400 IU/d) + Ca, 6mo	Improved symptoms and bone metabolism with supplementation
	Animal (dose ranging) [8]	Mouse celiac model	Cholecalciferol, villus/TJ, inflammation	High-dose vitamin D improved mucosal structure/TJs

AD, Atopic Dermatitis; PSO, Psoriasis; IBD, Inflammatory Bowel Disease; CD, Crohn’s Disease; UC, Ulcerative Colitis; CeD, Celiac Disease; EASI, Eczema Area and Severity Index; PASI, Psoriasis Area and Severity Index; SCORAD, SCORing Atopic Dermatitis; RCT, Randomized Controlled Trial; CDAI, Crohn’s Disease Activity Index; CRP, C-reactive protein; ESR, Erythrocyte Sedimentation Rate; GFD, Gluten-Free Diet; QOL, Quality of Life; MACE, Major Adverse Cardiovascular Events; BP, Blood Pressure; HOMA-IR, Homeostatic Model Assessment of Insulin Resistance. ↑ refers to an increase while ↓ refers to a decrease.

## Data Availability

Not applicable.
